# The role and therapeutic potential of cGAS-STING signaling in acute pancreatitis: from the perspectives of inflammation and cell death

**DOI:** 10.3389/fphys.2026.1976652

**Published:** 2026-10-26

**Authors:** Hanli Wang, Lingling Wen, Haiyang Wang, Yuxiang Liu, Zhizhun Mo

**Affiliations:** Emergency Department, Shenzhen Traditional Chinese Medicine Hospital, Shenzhen, Guangdong, China

**Keywords:** acute pancreatitis (AP), apoptosis, autophagy, cGAS-STING signaling, ferroptosis, pyroptosis

## Abstract

The cyclic GMP-AMP synthase (cGAS)-stimulator of interferon genes (STING) signaling pathway can recognize pathogen-derived and host DNA. This factor triggers innate immune responses and thereby establishes a broad and effective surveillance mechanism for tissue damage and pathogen invasion. In acute pancreatitis (AP), the cGAS-STING signaling pathway exerts diverse regulatory functions during the process of pancreatic cell death. We systematically review the molecular mechanism of the cGAS-STING signaling pathway in acute pancreatitis. We summarize the latest research progress on the interaction of this pathway with various forms of programmed cell death. Through specific case analyses, we elaborate on the key role of the cGAS-STING signaling pathway in regulating apoptosis, autophagy, ferroptosis, pyroptosis and inflammatory responses. Finally, this article also explores the potential research value of several natural plant-derived compounds. They achieve this by regulating this pathway and thereby interfering with the mechanism of cell death during the pathological process of acute pancreatitis.

## 1 Introduction

AP is a common inflammatory disorder (Trikudanathan et al., 2024). In severe cases, it can lead to pancreatic necrosis and other organ failure, with an overall mortality rate of approximately 1% to 5% (Petrov and Yadav, 2019). This disease not only results in significant short-term complications, such as pancreatic necrosis and organ dysfunction, but also contributes to long-term exocrine and/or endocrine insufficiency of the pancreas (Tenner et al., 2024). A subset of patients may develop recurrent episodes, prolonged functional impairment, and a reduced quality of life (Tenner et al., 2024). In clinical practice, the basic treatment is the same. Doctors will carry out fluid resuscitation and inhibit secretion for treatment. At the same time, they will administer pain relief and start enteral nutrition support as soon as possible (Szatmary et al., 2022). For severely ill patients, we need to implement more intensive interventions. We will transfer the patients to the ICU, administer antibiotics. At the same time, doctors will carry out endocrine replacement therapy and enhance energy support (Strum and Boland, 2023). However, patients with low blood volume, pancreatic necrosis, and systemic inflammatory response syndrome (SIRS) have a very high mortality rate. Moreover, AP has an extremely high recurrence rate and a risk of progressing to chronic pancreatitis. This also increases the risk of developing pancreatic cancer (Huang et al., 2024). It is primarily attributed to the repetitive injury inflicted on pancreatic tissue (Huang et al., 2024).

Ion channels and calcium signaling in acinar cells contribute to the initiation and progression of AP (Wang et al., 2024). Intrapancreatic activation of trypsinogen and subsequent autodigestion are considered key initiating events in AP (Gukovskaya et al., 2016; Zhao et al., 2018b; Mayerle et al., 2019; Chooklin and Chuklin, 2026).Various forms of cell death, such as autophagy, and pyroptosis, play crucial roles in disease progression, in addition significantly affect its clinical severity (Sendler et al., 2016). Since acinar cell injury is the central pathological event in AP, DAMPs, which activate the immune system and drive inflammatory responses (Zhou et al., 2024). During the process of cell death, the autoimmune response becomes more active due to the increased presence of nuclear DNA throughout the body (Zhou et al., 2024). Moreover, DNA sensors can detect extracellular DNA from dying acinar cells, thereby amplifying the inflammatory cascade and further worsens progression (Zhou et al., 2024).

Regulating inflammation to reduce cell death is a central therapeutic strategy in managing AP. The cGAS-STING signaling pathway regulates a wide range of biological processes in mammalian cells, including the induction of antiviral type I interferon (IFN- I) responses, production of pro-inflammatory cytokines, autophagy, cellular senescence, metabolic regulation, and apoptosis (Dvorkin et al., 2024). Disruption of cellular homeostasis can trigger abnormal cytoplasmic growth of DNA, which activates cGAS through identification of double-stranded DNA (dsDNA) (Zhang et al., 2025). Accumulating evidence suggests that the cGAS-STING pathway is a key of cytosolic DNA-induced IFN signaling (Bartok and Hartmann, 2020). However, its role in promoting AP pathogenesis remains incompletely understood. This review provides a comprehensive and critical synthesis of the current evidence on the involvement of cGAS-STING signaling in AP, with particular emphasis on its crosstalk with multiple forms of programmed cell death. Additionally, we explored its interactions with multiple cell death pathways. The ultimate goal of this study is to improve understanding of how the cGAS-STING pathway modulates inflammatory responses during pancreatic injury, thereby revealing its value in AP’s treating.

This review does not report new primary experimental data. Its contribution lies in integrating the currently available evidence on cGAS-STING signaling in AP, particularly its bidirectional crosstalk with apoptosis, autophagy, ferroptosis, and pyroptosis, and in discussing the therapeutic potential of natural plant-derived compounds targeting this axis.

## 2 Immune response in AP

The most widely accepted theory of the pathogenesis of AP describes it as an inflammatory disease. This is because the function of the acinar cells in secreting proteases is disrupted, resulting in excessive secretion, which subsequently triggers and sustains the inflammatory cascade reaction (Aney et al., 2024). The immune response plays a central role in disease progression. In the early stage, damage to acinar cells be set in a sterile environment, as the uninflamed pancreas is devoid of microbes (Mayerle et al., 2019). This injury increase the secretion of pro-inflammatory mediators, promotes immune cell infiltration, and induce sterile inflammation (Mayerle et al., 2019). Consequently, During the early phase of AP, the pancreatic injury occurs in a predominantly sterile environment, and immune cell recruitment and activation are primarily driven by DAMPs released from necrotic acinar cells, such as HMGB1, DNA, and ATP. However, in severe cases with intestinal barrier disruption, bacterial translocation and PAMP-mediated signaling can subsequently contribute to the amplification of systemic inflammation. Instead, necrotic pancreatic acinar cells release various damage-associated molecular patterns (DAMPs), such as high-mobility group box 1 (HMGB1), self-DNA, and other intracellular components (Kylänpää et al., 2010). These DAMPs activate pattern recognition receptors (PRRs) on infiltrating immune cells, inducing the production of additional inflammatory mediators (Kylänpää et al., 2010). This amplifies immune cell infiltration and intensifies the inflammatory response (Kylänpää et al., 2010). Accumulating evidence suggests that the SIRS in AP results from uncontrolled or dysregulated immune activation (Gukovsky et al., 2013).

A diverse array of immune cells infiltrates the pancreas shortly after the pathological process of AP, and their activity is closely linked to disease severity and clinical outcomes (Habtezion, 2015). These infiltrating immune cells include components of both the innate immune system—such as macrophages (Antonucci et al., 2015), neutrophils (Wan et al., 2019), dendritic cells (Bedrosian et al., 2011), mast cells (Lopez-Font et al., 2010), and natural killer cells (Shifrin et al., 2005)—and the adaptive immune response, including T (Son et al., 2019) and B lymphocytes (Son et al., 2019). Under normal conditions, immune cell infiltration acts that can promotes tissue repair and facilitates disease resolution. That is a protective mechanism. However, in some cases, if the initial injurious stimuli are not rapidly resolved, the immune-mediated inflammatory response may persist and intensify, potentially exacerbating pancreatic damage and triggering systemic inflammation (Sendler et al., 2018).

AP is initiated by the activation earlier than normal of trypsinogen and subsequent autodigestion of the pancreas. In some patients, the inflammatory process may extend beyond the pancreas, leading to a SIRS and, in severe cases, progressing to multiple organ dysfunction syndrome (MODS). During this progression, innate immune cells not only contribute to pancreatic injury but also interact with adaptive immune cells to form a sophisticated regulatory mechanism that as a coordinated role in both disease exacerbation and modulation (**Figure 1**).

**Figure 1:**
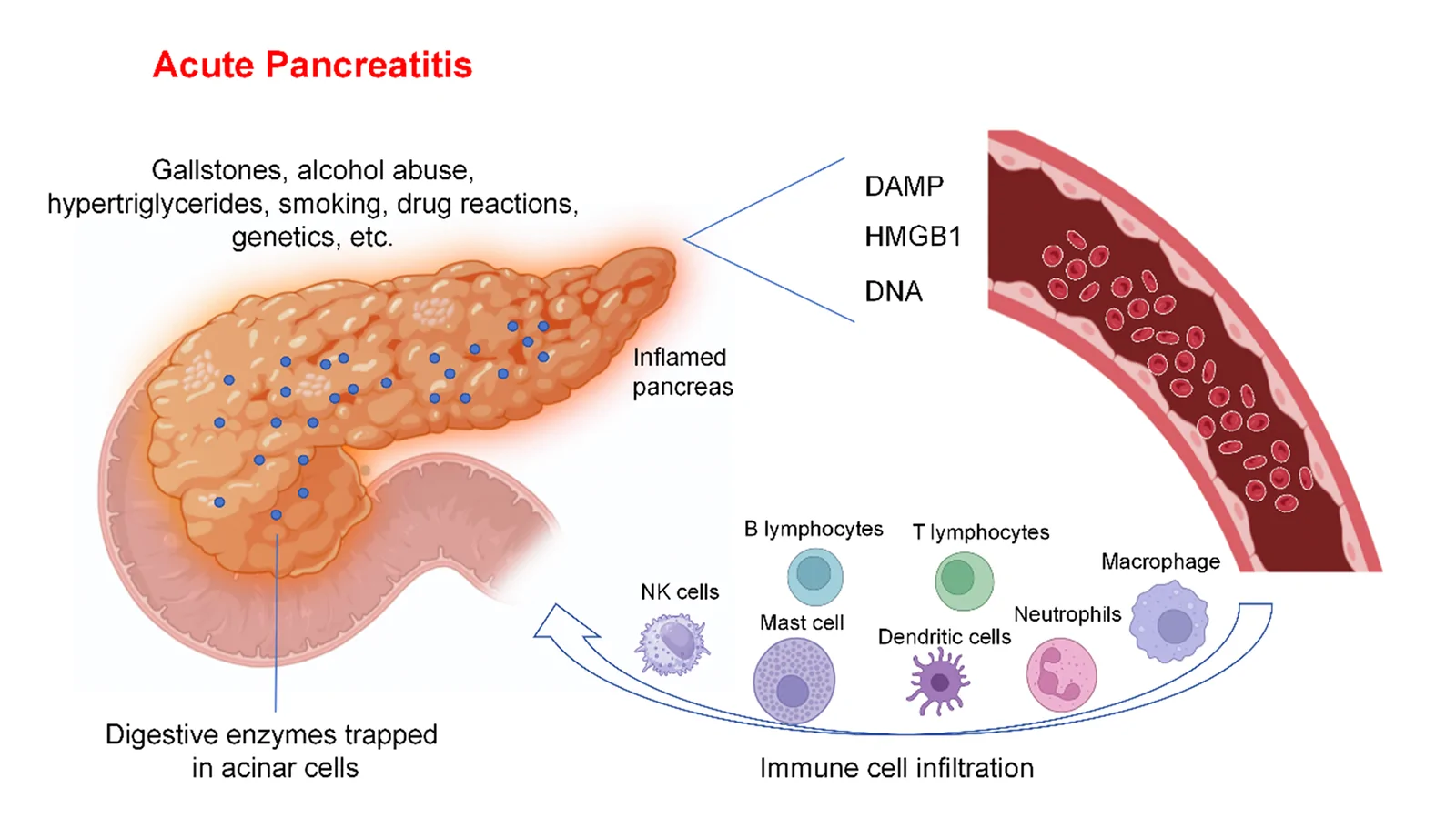
The immune response in AP.

## 3 cGAS-STING signaling pathway

The stimulate of innate immunity relies on the recognition of diverse pathogens, a process primarily mediated by the collaboration between PRRs and their ligands—PAMPs (Chen et al., 2025). Cytoplasmic DNA (cDNA) is a critical PAMP that can be detected by specific DNA sensors, thereby initiating downstream signaling in innate immune responses (Mankan et al., 2014). Among these, the cGAS-STING pathway plays a core play in the detecting of cDNA (Kwon and Bakhoum, 2020). Moreover, this pathway is also capable of sensing the release of self-DNA from dying cells, thus participating in the immune response to endogenous damage signals (Chen et al., 2016).

cGAS is a horseshoe-shaped protein mainly located in the cytosol (Civril et al., 2013). Structurally, it interacts with dsDNA through two cooperative binding sites, forming a 2:2 cGAS-DNA complex that is essential for its activation (Zhang et al., 2020). A unique zinc thumb motif allows cGAS to recognize B-form double-stranded DNA in a sequence-independent manner. DNA binding induces conformational changes and crosslinks two cGAS molecules to form a dimer; at higher DNA concentrations, cGAS can further assemble into higher-order oligomers and undergo liquid-liquid phase separation, which amplifies its catalytic activity. Following DNA binding, cGAS catalyzes the synthesis of the second messenger 2′3′-cGAMP from GTP and ATP. cGAMP then activates STING at the endoplasmic reticulum. Activated STING translocates to the Golgi apparatus, where it recruits TBK1 and phosphorylates IRF3. Phosphorylated IRF3 translocates to the nucleus and drives the transcription of type I interferon (IFN-I). In parallel, STING activates the NF-κB pathway, which promotes the expression of pro-inflammatory cytokines including IL-6 and TNF-α. Additionally, STING can activate STAT6 through TBK1-mediated phosphorylation at Ser407, an event independent of the JAK pathway (Du and Chen, 2018).

Following DNA binding, a structural change has occurred on cGAS, forming a ring-like structure that is capable of binding both GTP and ATP, thereby facilitating the synthesis of the second messenger 2′3′-cGAMP (Du and Chen, 2018). Subsequently, cGAMP functions as a signaling molecule, transmitting the activation signal to the downstream endoplasmic reticulum (ER) protein—stimulator of interferon genes (STING). After activation, STING moves from the ER to the Golgi apparatus. And it will activate TANK-binding kinase 1 (TBK1) in the Golgi apparatus, then phosphorylates interferon regulatory factor 3 (IRF3), initiating downstream signaling cascades. Activation of IRF3 downstream of TBK1 promotes the transcription of type I interferon (IFN-I). In parallel, STING-mediated activation of the NF-κB pathway drives the expression of pro-inflammatory cytokines such as IL-6 and TNF-α, which are distinct from the IRF3–IFN-I axis (Cheng et al., 2020). Upon binding to its receptor IFNAR1/2, IFN-I activates the Janus kinase (JAK)–signal transducer and activator of transcription (STAT) signaling pathway (Mathur et al., 2017). During this process, STAT1 and STAT2 are phosphorylated by the JAK kinase. Then it forms an interaction between the interferon regulatory factor 9 (IRF9) and the phosphorylated STAT1/2 complex. The combination of these two substances will generate the interferon-stimulated gene factor 3 (ISGF3) complex. And in the next biochemical reaction, it acts as a key transcription factor to trigger the expression of interferon-stimulated genes (ISGs) (Mesev et al., 2019).Moreover, STING can activate STAT6 through TBK1-mediated phosphorylation at Ser407, an event that is independent of the JAK pathway (Chen et al., 2011). Collectively, these detailed signaling events highlight the pivotal upstream regulatory role of cGAS in the STING.

In addition to inducing IFN-I responses, activation of STING can also trigger the nuclear factor-kappa B (NF-κB) signaling pathway (Hopfner and Hornung, 2020). Recent studies have demonstrated that TBK1 and IκB kinase epsilon (IKKϵ) exhibit functional redundancy, enabling them to phosphorylate STING and promote the recruitment and activation of the transforming growth factor β-activated kinase 1 (TAK1) and its associated IκB kinase β (IKKβ) complex. This process results in the phosphorylation and subsequent degradation of IκB inhibitory proteins, thereby releasing the NF-κB (p65/p50) dimer and facilitating its translocation into the nucleus to activate the NF-κB (Balka et al., 2020). It has been reported that the classical NF-κB regulates microtubule-dependent transport mechanisms, allowing STING to be transported to the lysosome, which prevents its degradation and enhances STING-mediated signaling (Zhang et al., 2023). In contrast, activation of the non-conventional NF-κB has been associated with reduced expression of type I interferons (Hou et al., 2018). These findings suggest that the conventional and non-conventional NF-κB pathways exert opposing regulatory effects on STING signaling (**Figure 2**).

**Figure 2:**
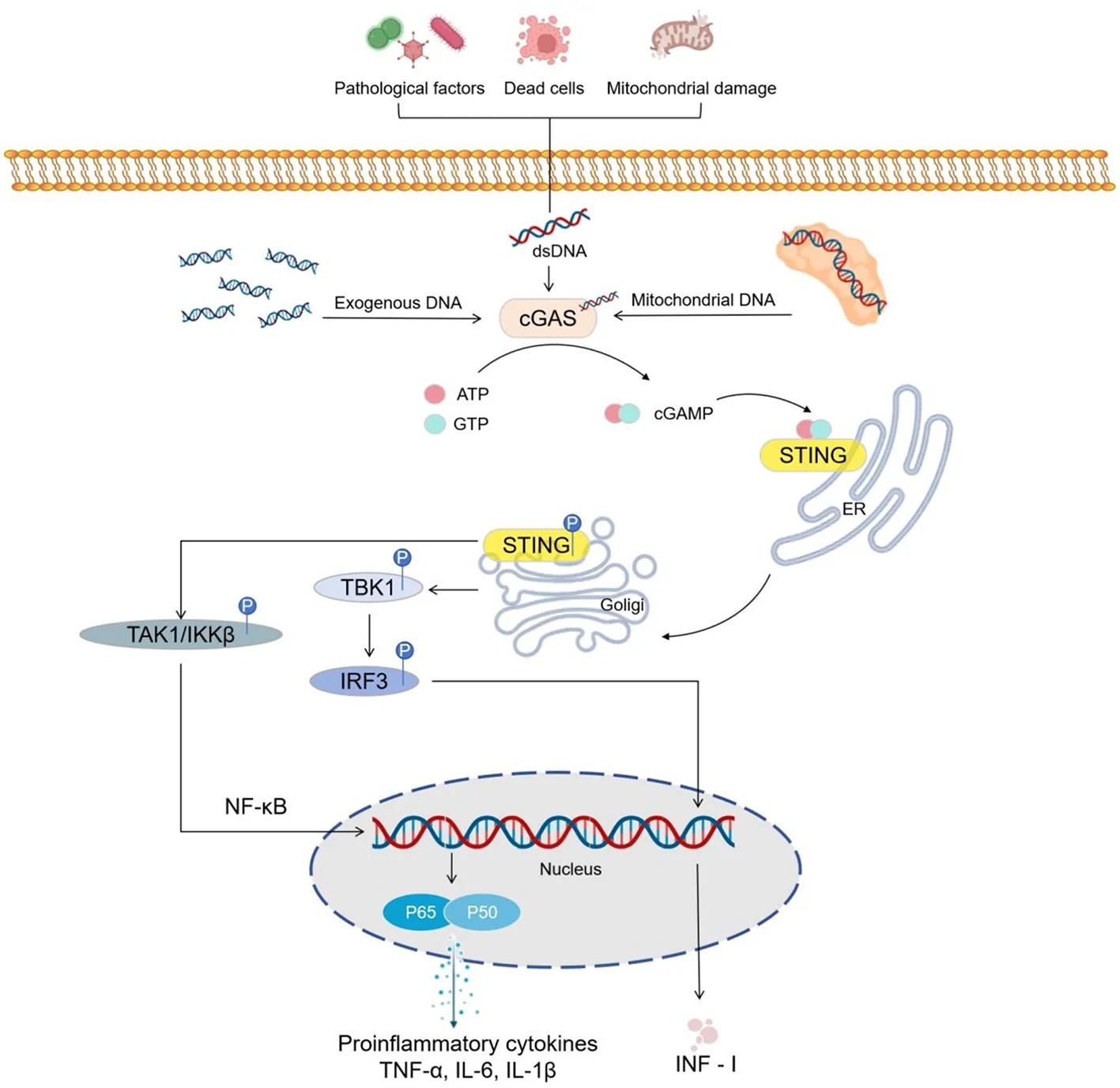
The activation mechanism of the CGAS-STING signaling pathway.

## 4 The role of the cGAS-STING signaling pathway in AP

Pancreatitis is a prevalent disorder, classified into acute and chronic forms based on its progression (Habtezion et al., 2019). The pathomechanism of AP is pancreatic acinar cell death and a robust inflammatory response. When AP progresses to severe acute pancreatitis (SAP), it is associated with a significantly increased mortality rate (Watanabe et al., 2017). During the pathogenesis of AP, acinar cell death, along with systemic and localized inflammation, represents key pathological events (Gukovskaya et al., 2016). Damaged acinar cells secrete cytokines, chemokines, and inflammatory mediators, which promote the recruitment and infiltration of granulocytes to the site of injury (Criddle et al., 2007). As immune cells migrate toward the pancreas, intracellular contents from damaged acinar cells are released into the extracellular environment. These endogenous molecules play a crucial role in activating immune cells—particularly macrophages—and directing their chemotaxis, thereby intensifying the inflammatory cascade (Mihoc et al., 2024). In severe cases of AP, pancreatic tissue necrosis and acinar cell death critically influence disease onset and progression. Studies have demonstrated that DNA released into the circulation can serve. Dysregulated or impaired autophagic flux, rather than increased autophagy per se, has been implicated in acinar cell injury and the amplification of inflammatory responses in AP [16.42.79]. Cytoplasmic self-DNA have been demonstrated to activate the STING and contribute to the pathogenesis of certain autoimmune diseases (Gall et al., 2012). In murine models of AP, STING senses acinar cell death and activates pro-inflammatory signaling pathways (Zhao et al., 2018a). Macrophages express STING and actively participate in the initiation and amplification of pancreatic inflammation (Zhao et al., 2018a). Clinical evidence further supports that mitochondrial DNA (mtDNA) leakage can exacerbate acinar cell injury via the cGAS-STING-NF-κB axis (Zhang et al., 2025). It has been established that interleukin-1 (IL-1), IL-6, and tumor necrosis factor (TNF) can induce acinar cell damage and trigger mitochondrial stress responses (Mititelu et al., 2024). Upon transcription and release into the extracellular space, pro-inflammatory cytokines activated by the STING pathway can act directly on acinar cells, leading to more severe mitochondrial stress and mtDNA leakage. This, in turn, further enhances STING pathway activation and cytokine production, forming a positive feedback loop. Which may contribute to the initiation and progression of AP (Zhang et al., 2025).

In SAP, secondary infection represents contributor to the high mortality rate (Petrov et al., 2010). In a rat model of SAP, enhanced pathological responses in both the pancreas and intestine were observed, accompanied by upregulation of STING-related proteins and genes. Notably, inhibition of STING signaling was shown to alleviate intestinal epithelial cell (IEC) damage following SAP (Zhang et al., 2023). These findings are supported by related studies: in an intestinal injury model of SAP, expression of the cGAS-STING pathway was elevated, and its modulation improved intestinal function in mice (Li et al., 2023). Furthermore, suppression of HDAC3 through regulation of the cGAS-STING pathway in intestinal glial cells was found to ameliorate AP in murine models (Li et al., 2023). In a study focusing on SAP complicated with acute lung injury (SAP-ALI), it was demonstrated that the mtDNA-cGAS-STING axis promoted NLRP3 inflammasome-mediated pyroptosis of macrophages by enhancing the nuclear translocation of phosphorylated IRF3 and increasing IRF7 expression levels (Peng et al., 2024). Further experimental evidence revealed that the release of mitochondrial DNA (mtDNA) facilitated the infiltration of inflammatory monocytes in SAP-ALI via the STING/TLR9-NF-κB-JMJD3-TNF-α signaling pathway (Chen et al., 2023).

Research has found that the STING pathway plays opposite roles in different types of pancreatitis. Zhao et al. demonstrated that it aggravates the pathological process of acute pancreatitis by promoting the release of TNFα and IFNβ (Zhao et al., 2018a); while in chronic pancreatitis, this pathway exerts a certain protective effect by regulating the infiltration of Th17 cells (Zhao et al., 2019). Unlike acute pancreatitis, the activation of STING shows a protective effect in chronic pancreatitis, which may be due to the fact that the STING signal regulates different immune cell subsets in different pathological states or in inflammatory environments (Zhao et al., 2019).

The islets of Langerhans have endocrine functions and mainly secrete hormones, that encompasses insulin and glucagon. The islets are composed of various cells, among which β cells are responsible for the synthesis and release of insulin (Benninger and Kravets, 2022). Interestingly, studies have found that STING is significantly expressed in both animal and human pancreatic β cells (King et al., 2019), and it has been reported that the STING is closely related to certain β cell injuries (Hu et al., 2020). Lipotoxicity injury of pancreatic β cells is one of the important pathological features of type 2 diabetes (Ye et al., 2019). In addition, some studies suggest that the STING may affect the occurrence and development of diabetes by inhibiting the activity of diabetogenic T cells and regulating the integration and secretion of insulin (Akazawa et al., 2021).In summary research results, it can be considered that the cGAS-STING pathway has extensive and diverse biological effects in the pancreas (**Figure 3**).

**Figure 3:**
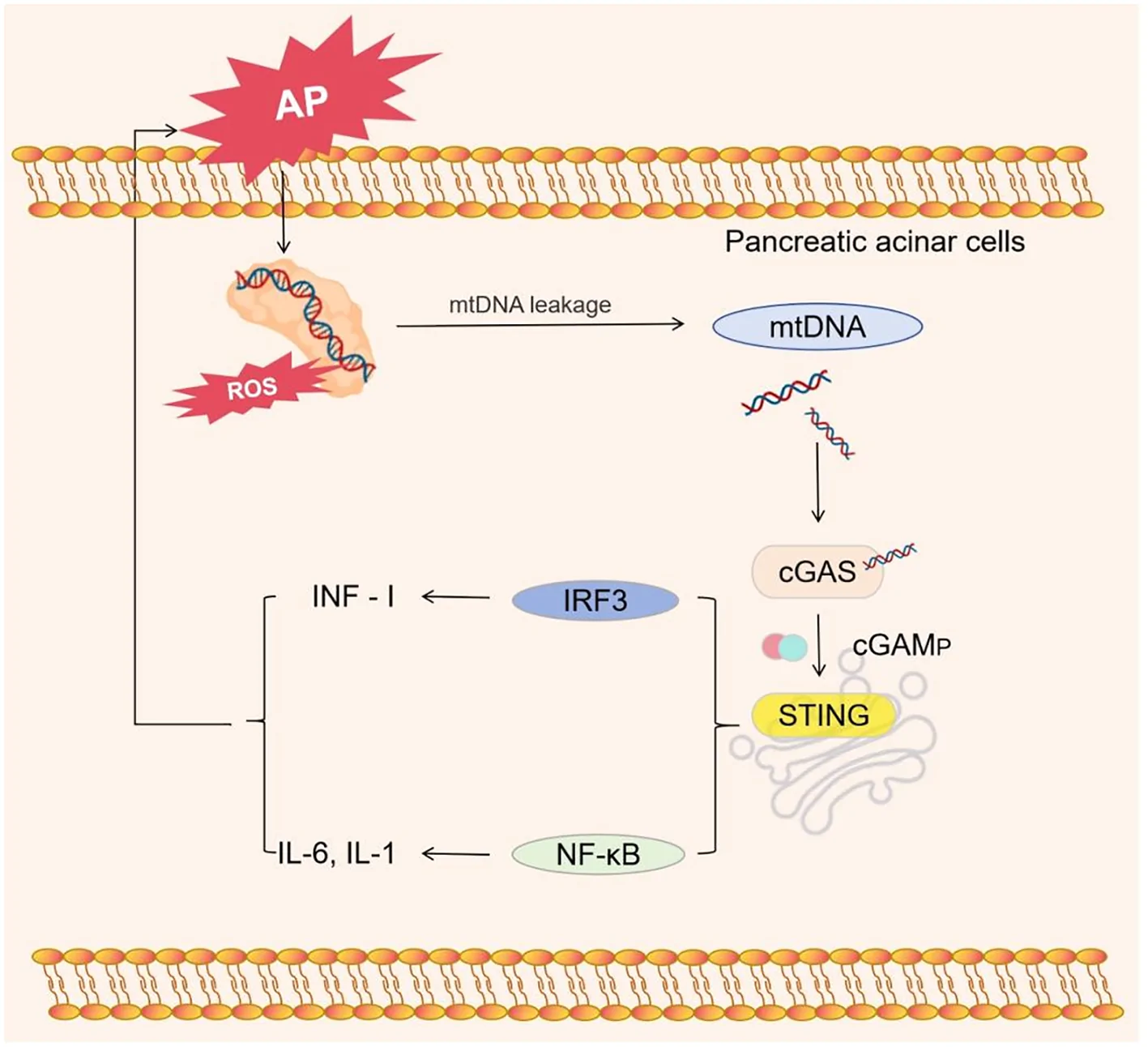
The role of the cGAS-STING signaling pathway in AP.

## 5 The interaction between the cGAS-STING pathway and various forms of cell death

The pathogenesis of AP remains incompletely understood, and currently, no specific treatment are available. Accumulating evidence indicates that multiple immune cell types can infiltrate the affected pancreatic tissue in SAP and may play pivotal roles in disease progression. In-depth characterization of the AP immune microenvironment has revealed dysregulated activity in key signaling pathways, including apoptosis, oxidative stress, autophagy, ferroptosis, and inflammatory responses (Fang et al., 2022). This section provide a systematic overview of the mechanistic roles of these signaling pathways and their potential crosstalk with the cGAS-STING pathway.

### 5.1 The role of the cGAS-STING pathway in apoptosis in AP

In recent years, numerous studies have focused on the mechanisms by which the cGAS-STING pathway induces apoptosis and its associated biological consequences (Zheng et al., 2023). Apoptosis, the earliest identified form of programmed cell death, primarily occurs through extrinsic and intrinsic pathways (Nössing and Ryan, 2023). These two pathways ultimately converge at mitochondrial outer membrane permeabilization (MOMP), leading to the release of cytochrome c and other regulatory molecules, activation of the caspase cascade, and ultimately, execution of cell death. Genetic ablation of STING consistently attenuates AP severity across cerulein- and diet-induced models, as evidenced by reduced pancreatic edema, lower serum lipase, and decreased neutrophil infiltration (Tang et al., 2019). Bone marrow chimera experiments further localize the pathogenic function of STING to infiltrating leukocytes—predominantly macrophages—rather than resident acinar cells. Pharmacological STING inhibition recapitulates the protective phenotype of genetic deletion, supporting a causal, rather than correlative, role for this pathway. The balance between pro-apoptotic and anti-apoptotic members of the B-cell lymphoma-2 (Bcl-2) family—such as Bax, Bak, Bcl-2, and Bcl-xL—plays a decisive role in regulating MOMP (Singh et al., 2019). The formation of the Bax/Bak complex controls the MOMP, facilitating the release of mitochondrial contents, including mtDNA, which serves as a potent activator of cGAS in the cytoplasm (Cosentino et al., 2022). This release may also be synergistically influenced by mitochondrial inner membrane permeabilization (Riley et al., 2018). Conversely, caspase-3 and caspase-7, which are activated during apoptosis, can suppress the activity of the mtDNA-cGAS-STING in both human and murine cells by cleaving cGAS and IRF3 (Ning et al., 2019). In the murine model of AP, acinar cell death has been confirmed to bring about to disease progression through this mechanism (Gomez et al., 2001).

The cGAS-STING pathway promotes apoptosis through various mechanisms. The most direct way is the activation of STING. Some researchers have reproduction that in model of AP, exosomes (M1-EVs) derived from M1-type macrophages carrying inflammatory mitochondria can enter pancreatic β cells. These affected mitochondria fuse with the mitochondria of the cells item mentioned above, causing lipid peroxidation and mitochondrial structural damage, leading to the release of mtDNA into the cytoplasm and subsequently activating the STING, ultimately inducing apoptosis (Gao et al., 2024). Additionally, the cGAS-STING pathway enhances the apoptotic effect by inducing the production of IFN- I, up-regulating the abundance of pro-apoptotic protein NOXA, and simultaneously down-regulating the expression of anti-apoptotic protein Bcl-xl (Lohard et al., 2020). BCL2 has been confirmed as an important biomarker of immunogenic cell death in severe acute pancreatitis (Wang et al., 2023), but the specific role of STING in this process still requires further research. In an AP rat model, the levels of STING and its downstream molecule IRF3 are positively correlated with the severity of inflammation. Moreover, cGAS can recognize chromatin fragments in the cytoplasm during mitotic arrest, bring about the phosphorylation and accumulation of IRF3. This phosphorylated IRF3 can interact with anti-apoptotic protein Bcl-xl and inhibit its function, thereby promoting MOMP and triggering apoptosis in various cell lines (Zierhut et al., 2019). Abnormal T cell function (Zhou et al., 2020) and calcium homeostasis imbalance (Zhang et al., 2024) are important pathological features of acute pancreatitis. In human and mouse cells, the continuous activation of STING can disrupt calcium homeostasis within T cells, making them highly sensitive to endoplasmic reticulum stress and unfolded protein response (UPR).And these reactions are achieved through T cell receptor signaling, ultimately leading to T cell death (Wu et al., 2019).

We also noticed that it was mentioned in some literature that interpretation of cGAS-STING data in AP must account for model-dependent heterogeneity. Cerulein-induced AP primarily models the early, edema-predominant phase and is characterized by mild-to-moderate severity. In contrast, sodium taurocholate-induced AP produces extensive pancreatic necrosis and a more robust systemic inflammatory response, whereas L-arginine administration induces a necrotizing phenotype with distinct acinar cell death kinetics (Chooklin and Chuklin, 2026). Whether cGAS-STING functions equivalently across these models—or whether its contribution is weighted toward specific severity strata—remains to be systematically evaluated. Notably, the majority of available mechanistic evidence derives from cerulein-based models, which may underrepresent the pathway’s role in necrotizing SAP (Huang et al., 2026).At the same time, we have noticed that in some literature, it has been mentioned that cell-type-resolved analysis reveals a consistent pattern: STING exerts pro-inflammatory effects primarily through infiltrating macrophages, where it senses DNA released from dying acinar cells and drives TNF-α and IFN-β production. In contrast, the functional significance of STING in pancreatic acinar cells, islet β cells, and ductal/stellate cells remains less well defined. Conditional knockout models targeting STING in specific pancreatic cell populations would be necessary to disentangle cell-autonomous from paracrine contributions (Zhao et al., 2018b) (**Figure 4**).

**Figure 4:**
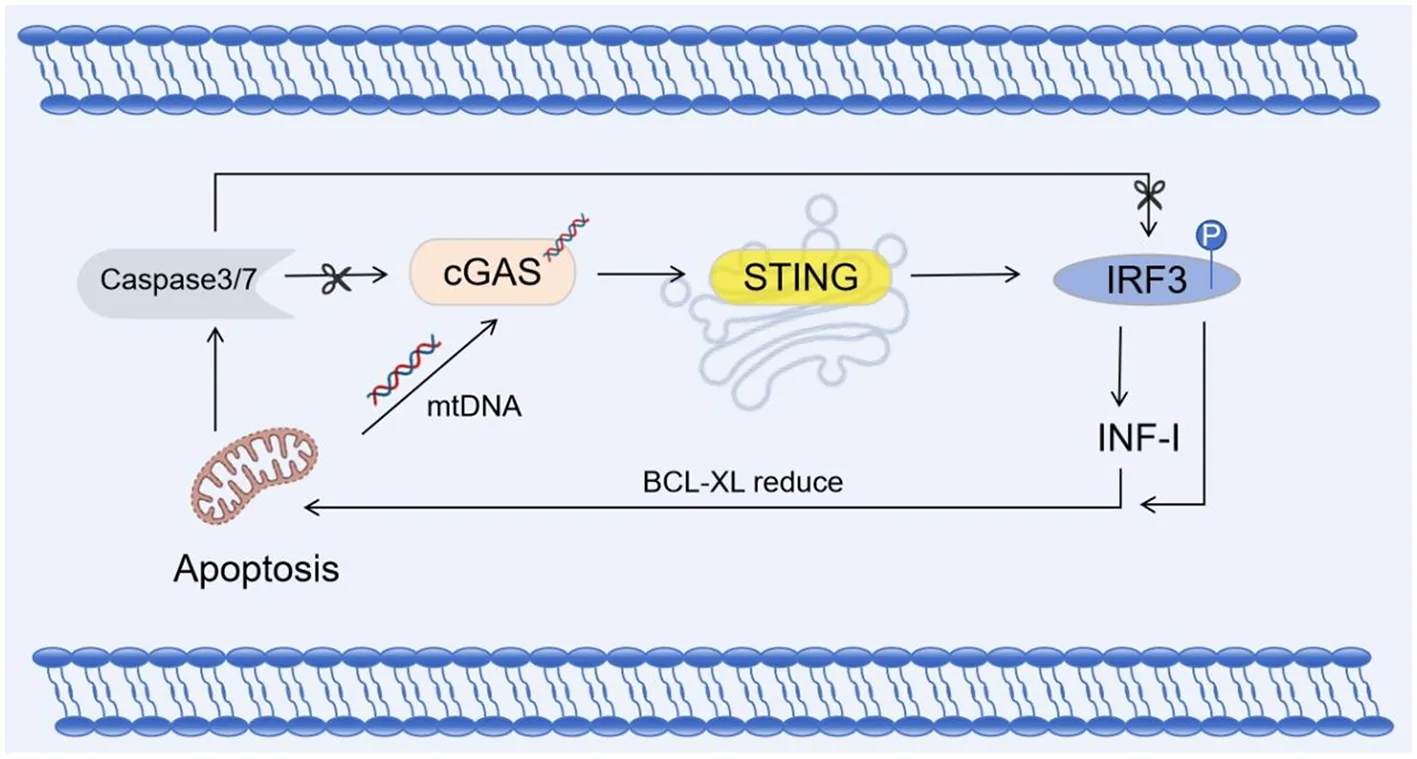
The role of the cGAS-STING pathway in apoptosis in AP.

### 5.2 The role of the cGAS-STING pathway in autophagy in AP

Accumulating evidence has demonstrated that autophagy play critically role in the pathogenesis of AP (Hashimoto et al., 2008). As early as 70 years ago, researchers observed the accumulation of autophagic vacuoles and extensive acinar cell damage in human AP tissue samples (Helin et al., 1980). Cytoplasmic vacuolization represents one of the earliest morphological changes in pancreatic tissue injury and is often closely associated with alterations in zymogen granules and the formation of autophagosomes (Helin et al., 1980). A study by O. Mareninova and colleagues further revealed that autophagy is impaired in AP and proposed that vacuolization and premature trypsinogen activation may result from blocked or delayed autophagic flux (Mareninova et al., 2009). In recent years, increasing evidence has indicated that pathological vacuolization indeed reflects the abnormal accumulation of autophagosomes (Ohmuraya and Yamamura, 2008). GFP-LC3, a widely utilized marker of autophagy, serves as a critical tool in autophagy research, particularly in the context of the pancreas, through the use of transgenic GFP-LC3 models (Mizushima et al., 2004). Moreover, other autophagy-related proteins, such as ATG5 (Diakopoulos et al., 2015) and ATG7 (Xia et al., 2020), have also been implicated in the development of acute pancreatitis. The autophagy observed in AP represents a dysregulated autophagic process that may contribute to disease exacerbation. As autophagy levels increase, the inflammatory response intensifies, thereby worsening the pathological progression of pancreatitis (Kocsis et al., 2009).

The cGAS-STING-induced autophagy plays a key role in preventing excessive immune activation and sustained immune stimulation (Corrales et al., 2016). This pathway modulates the innate immune response through multiple mechanisms, including the induction of autophagy, the degradation of cGAS and STING proteins, and the clearance of cytoplasmic DNA and pathogens. cGAS initiates autophagy by interacting with LC3 and Beclin-1, it suppressing the synthesis of 2′3′-cGAMP and blocking IFN- I production, in addition promoting the autophagy-mediated degradation of cGAS and cytoplasmic DNA, which prevents overactivation of cGAS and prolonged immune signaling (Liang et al., 2014). STING can enter the autophagy pathway via TBK1-mediated phosphorylation of p62, leading to its ubiquitination and following autophagic decomposition, thus attenuating sustained activation of innate immune signaling (Wu and Lu, 2019). In AP, autophagy occurs in pancreatic acinar cells and is accompanied by activation of the STING-TBK1 pathway (Song et al., 2021). STING is mediated by cytosolic DNA through the DNA sensor cGAS, which subsequently recruits TBK1. TBK1 then trigger NF-κB via a mechanism governed by TRAF6 and can also directly phosphorylate and activate IRF3. Ultimately, the activation of this pathway led to the production of pro-inflammatory factors such as TNFα and IFNβ. Therefore, autophagy, as a key intracellular defense mechanism, can sequester invading pathogens in the cytoplasm and direct them toward lysosomal degradation, thereby effectively limiting infection and maintaining cellular homeostasis (Gutierrez et al., 2004) (**Figure 5**).

**Figure 5:**
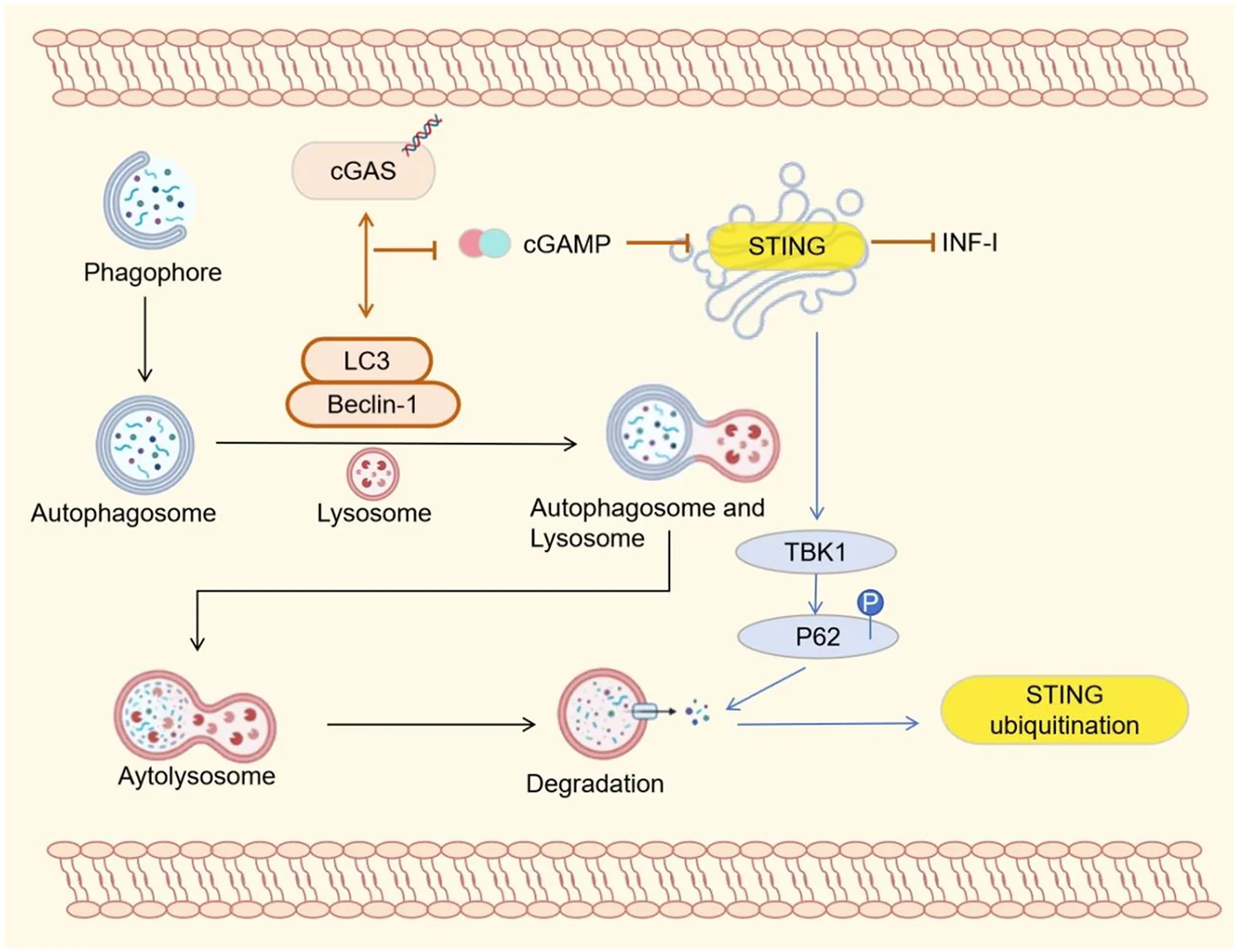
The role of the cGAS-STING pathway in autophagy in AP.

### 5.3 The role of the cGAS-STING pathway in ferroptosis in pancreatitis

Ferroptosis is essential difference from apoptosis in terms of genetic, morphological, and biochemical characteristics (Qiu et al., 2020). The factors related to the pathophysiological process of this disease include excessive reactive oxygen species (ROS), uncontrolled iron metabolism and excessive lipid oxidation. These characteristic features play a crucial role in it (Kimita and Petrov, 2020). These observations suggest that ferroptosis may play a crucial role in the initiation and progression of AP. For example, research by Dai demonstrated that mice fed a high-iron diet exhibited significantly elevated pancreatic iron levels and were more susceptible to AP compared to those maintained on a normal diet (Dai et al., 2020). Moreover, specific knockout of the GPX4 gene in the pancreas of mouse models markedly exacerbated pancreatic damage induced by cerulein or L-arginine administration. Notably, this pathological deterioration could be effectively mitigated by the ferroptosis inhibitor liproxstatin-1, indicating that iron overload and reduced glutathione peroxidase 4 (GPX4) expression contribute to the progression of experimental pancreatitis through the induction of oxidative damage. Further microarray analysis revealed that in the AP model, NOX2 expression was upregulated while GPX4 expression was downregulated, with their expression levels positively correlated with disease severity (Chen et al., 2025).

The amino acid transporter system Xc^-^ (System Xc^-^) functions as a cystine/glutamate antiporter. Intracellular cystine is reduced to cysteine, which serves as a critical precursor for the biosynthesis of glutathione (GSH). GPX4, plays a central role in the regulation of ferroptosis. In the presence of GSH, GPX4 catalyzes the reduction of free H_2_O_2_ or organic peroxides (ROOH) to water or corresponding alcohols, respectively (Priyanka et al., 2025). Depletion of GSH elicit to the inactivation of GPX4, contribute to the accumulation of lipid ROS and the induction of ferroptosis. The relationship between GPX4-dependent ferroptosis and cGAS-STING signaling is bidirectional and context-dependent. In the presence of sufficient GPX4 activity, STING signaling proceeds normally; however, GPX4 deficiency or inactivation promotes STING carbonylation, impairs its ER-to-Golgi trafficking, and suppresses downstream IFN-I production (Jia et al., 2020). Conversely, sustained STING activation can stabilize STING dimers and interact with NCOA4 to promote ferritinophagy, thereby amplifying lipid peroxidation and ferroptosis (Wang et al., 2025). In the AP context, the net effect likely depends on the temporal sequence and magnitude of each arm: early mitochondrial damage and GPX4 loss may transiently suppress STING signaling through carbonylation, while persistent mtDNA release from necrotic acinar cells could subsequently drive STING-NCOA4-mediated ferroptosis in macrophages. The relative dominance of these opposing mechanisms has not been experimentally resolved and represents a priority for future investigation. In SAP, abnormal expression of NCOA4 has been observed, and targeting NCOA4 N-glycosylation can inhibit ferritinophagy-mediated, autophagy-dependent ferroptosis in combination with FTH1, thereby ameliorating the pathological features of SAP (Lu et al., 2025). Currently, most studies on ferroptosis focus on pancreatic cancer, and research specifically addressing its role in acute pancreatitis remains limited. A study on pancreatic ductal cancer revealed that GPX4 deficiency trigger the TMEM173/STING-dependent DNA sensing pathway, leading to macrophage infiltration and activation, and promoting Kras-driven PDAC progression (Del Poggetto et al., 2021). In summary, ferroptosis and the cGAS-STING pathway are closely interconnected, and the aforementioned findings provide both a theoretical framework and experimental support for further investigation during pancreatitis.

It should be emphasized that the evidence linking GPX4 deficiency to STING-dependent DNA sensing and macrophage infiltration derives primarily from pancreatic ductal adenocarcinoma (PDAC) models. In PDAC, GPX4 loss triggers TMEM173/STING-dependent DNA sensing that promotes tumorigenesis. While these findings provide a mechanistic template, the extrapolation to AP is non-trivial: AP is an acute inflammatory process driven by acinar cell necrosis, whereas PDAC involves chronic epithelial transformation and stromal remodeling. The role of GPX4-STING crosstalk in acute pancreatic injury remains to be directly demonstrated (**Figure 6**).

**Figure 6:**
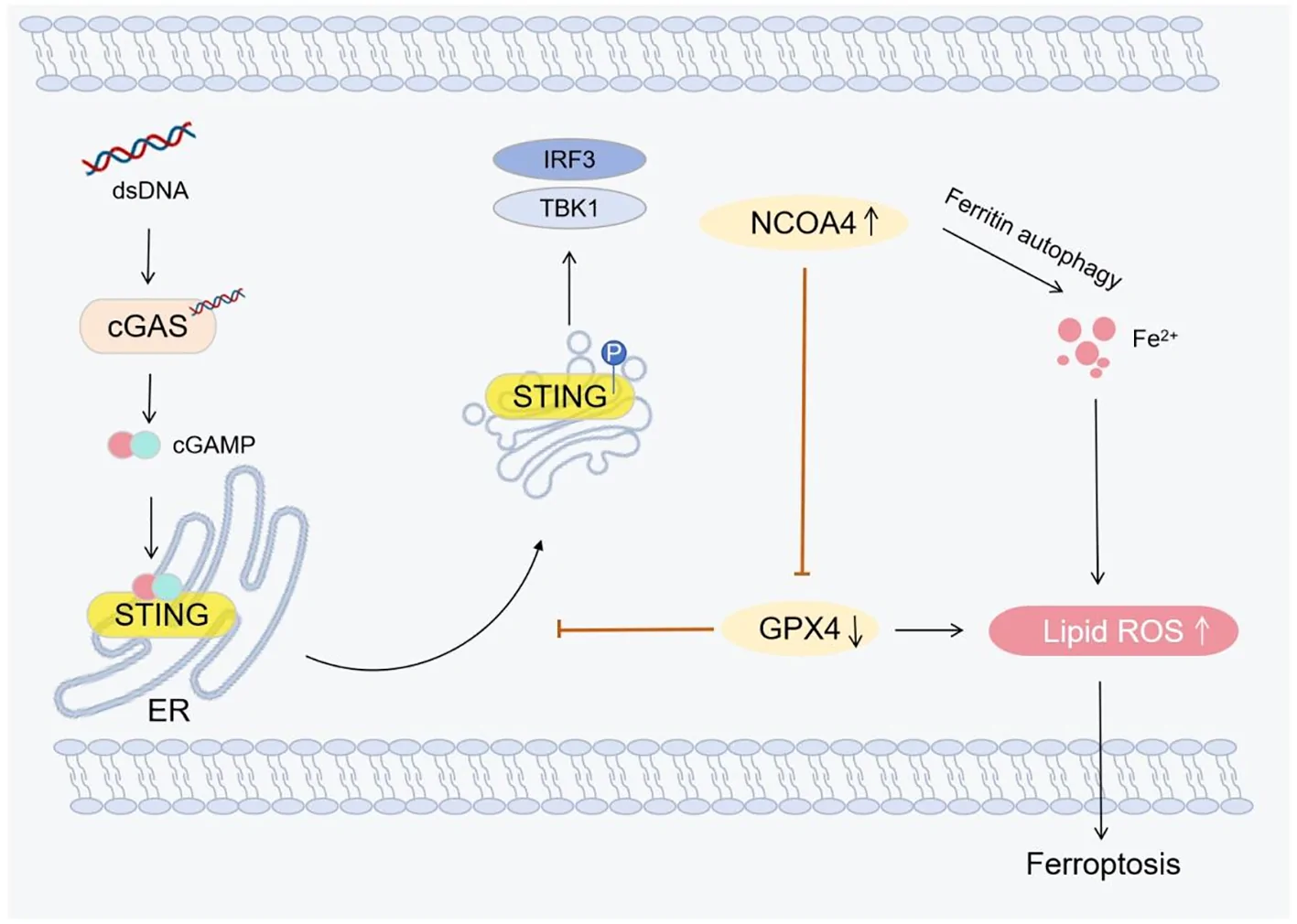
The role of the cGAS-STING pathway in ferroptosis in AP.

### 5.4 The role of the cGAS-STING pathway in pyroptosis in pancreatitis

Pyroptosis where it manifests as a distinct of programmed cell death which caspase-1-dependent (Fink and Cookson, 2006). Inflammasomes are primarily composed of Nod-like receptor (NLR) family members, including Multiple NLR receptors and AIM2, the adaptor protein apoptosis-associated speck-like protein (ASC), and the pro-form of caspase-1 (pro-caspase-1) (Martinon et al., 2002). Upon detection of pathological disturbances in the cytoplasm, pro-caspase-1 and ASC associate with NLRP3 or AIM2 to form inflammasome complexes. This leads to the activation and cleavage of pro-caspase-1. Caspase-1 not only Stimulates the conversion of pro-inflammatory cytokines into their active forms such as pro-IL-1β and pro-IL-18 but triggers the activation of gasdermin D (GSDMD) through proteolytic cleavage as well (Ramirez et al., 2018). The N-terminal fragment of GSDMD oligomerizes to form membrane pores, ultimately resulting in the release of inflammatory mediators, cellular swelling, and plasma membrane rupture (Martinon et al., 2002). In an *in vitro* AP injury model using AR42J cells, typical ultrastructural changes and activation of the NLRP3/caspase-1 were observed, further confirming the occurrence of pyroptosis in acinar cells (Xia et al., 2019). Notably, activated caspase-1 can cleave cGAS, thereby suppressing the FN response to viral infection (Chen et al., 2024). In contrast, GSDMD pore formation promotes potassium efflux, and the resulting depletion of intracellular K^+^ is necessary and sufficient to restrain cGAS-dependent type I interferon responses. This represents a negative regulatory mechanism by which pyroptosis limits sustained cGAS-STING activation (Wang et al., 2017). Additionally, LPS-induced mtDNA into the cytoplasm enhances cGAS-STING pathway (Banerjee et al., 2018). These findings collectively indicate that pyroptosis can either exerts bidirectional regulate on the cGAS-STING pathway depending on the cellular context. Moreover, the cGAS-STING pathway further amplifies NLRP3 inflammasome-mediated pyroptosis through IRF3-dependent upregulation of NLRP3 expression (Ning et al., 2020). The involvement of NLRP3 in AP has been extensively validated. STING directly interacts with NLRP3, facilitating its recruitment to the endoplasmic reticulum and reducing its polyubiquitination, thereby enhancing inflammasome activation and the inflammatory response to infection (Ferrero-Andrés et al., 2020; Lyu et al., 2024). Guanylate-binding proteins (GBPs), which are interferon-stimulated gene (ISG) products induced by IFN, promote the activation of caspase-family by mediating the release of bacterial components into the cytoplasm (Liu et al., 2018). Furthermore, in macrophages, GBPs can induce DNA release to activate the cGAS-STING pathway, thereby triggering the IFN response and enhance pyroptosis (Lyu et al., 2024). Excessive apoptosis has been confirmed to be a key link in the pathophysiological process of inflammatory diseases such as sepsis (Voet et al., 2019).

Inflammatory injury to pancreatic acinar cells (PACs) is a key initiating event in the process of acute pancreatitis. Chen et al. demonstrated that Saikosaponin D protects pancreatic acinar cells against cerulein-induced pyroptosis by alleviating mitochondrial damage and inhibiting the cGAS-STING pathway (Chen et al., 2024). Furthermore, acute lung injury (ALI) is a complication arising from the widespread inflammation associated with severe acute pancreatitis (SAP) and represents one of common extrapancreatic manifestations of the disease (Wang et al., 2022). In studies on ALI caused by SAP, damaged mitochondria release mtDNA into the cytoplasm, a process triggered by the activation of macrophages. The mtDNA-cGAS-STING axis upregulates IRF7 levels, with the upstream mechanism being phosphorylated IRF3, and it participates in the NLRP3 inflammasome-mediated pyroptosis process. Various interventional strategies, including mitochondrial depletion, cGAS gene knockout or pharmacological inhibition, and suppression of IRF3 or IRF7 expression, have been shown to modulate NLRP3 inflammasome activation, thereby reducing the severity of macrophage pyroptosis and SAP-induced ALI (Peng et al., 2024).

### 5.5 Integrative model and unresolved questions

Synthesizing the available evidence, we propose that cGAS-STING functions as both a driver and a consequence of AP pathogenesis, with the following causal architecture: (1) Initiation phase: Acinar cell injury—triggered by trypsinogen activation, calcium overload, or bile acid exposure—induces mitochondrial dysfunction and mtDNA release. This represents the upstream driver event. (2) Amplification phase: Extracellular mtDNA and nuclear DNA from dying acinar cells activate cGAS-STING in infiltrating macrophages, leading to IFN-I and NF-κB-dependent cytokine production. This step converts a localized acinar injury into a systemic inflammatory response. (3) Feedback phase: Pro-inflammatory cytokines (TNF-α, IL-6) act back on acinar cells to induce further mitochondrial stress and mtDNA leakage, establishing a self-amplifying loop that sustains cGAS-STING activation. (4) Resolution or progression: Depending on the magnitude and duration of mtDNA release, cGAS-STING may either promote tissue repair through autophagy-mediated clearance of cytosolic DNA, or drive persistent inflammation and multi-organ injury through ferroptosis and pyroptosis.

The key unresolved question is whether cGAS-STING activation is a sufficient driver of AP progression or merely an amplifying consequence of acinar cell death. Genetic epistasis experiments—comparing the severity of AP in mice with acinar-specific vs. myeloid-specific STING deletion—would be required to formally distinguish these possibilities.

## 6 The potential value of natural plant-derived compounds targeting the cGAS-STING pathway in regulating cell death in AP

Although the therapeutic potential of cGAS-STING agonists and antagonists in AP has been discussed in previous reviews, the present review specifically focuses on bioactive natural plant-derived compounds and their modulatory effects on AP-related cell death, with an emphasis on the cGAS-STING pathway.

Saikosaponin D (SSd), a triterpene saponin isolated from the traditional Chinese medicinal herb Bupleurum chinense, Demonstrated pharmacological activity against inflammation diseases (Wu et al., 2022). there is growing evidence that SSd can attenuate cell injury of AP induced by cerulein by inhibiting NLRP3/caspase-1-mediated pyroptosis (Ramirez et al., 2018). Notably, SSd reduces mitochondrial damage, decreases oxidative stress levels, and suppresses the release of mitochondrial DNA (mtDNA), thereby inhibiting the activation of the cGAS-STING pathway. From a mechanistic perspective, SSd has also been shown to inhibit the activation of the MAPK in pancreatic acinar cells, further contributing to its protective effects (Li et al., 2021).

Artemisinin, a sesquiterpene lactone isolated from the traditional Chinese medicinal herb, has been shown to reduce the abnormal activation of trypsinogen, inhibit neutrophil activation, and induce apoptosis in pancreatic acinar cells. These researches suggest that artemisinin can trigger intrinsic apoptosis via the caspase-3 signaling pathway, thereby exerting protective effects against AP (Zhao et al., 2007). During apoptosis, caspase-3 or caspase-7 cleaves cGAS and IRF3, which suppresses the activation of the mtDNA-mediated cGAS-STING pathway (White et al., 2014). Studies have demonstrated that the artemisinin derivative artesunate can exert neuroprotective effects by inhibiting cGAS-STING pathway-mediated inflammation. However, its functional role in pancreatic tissue remains to be further elucidated (Wei et al., 2025).

Kaempferol has been shown to inhibit the STING/NLRP3 signaling pathway in adipose tissue, and in acute lung injury models it suppresses cGAS-STING activation by restoring mitochondrial membrane potential and reducing cytosolic DNA release. However, these studies were conducted in metabolic and pulmonary contexts, and direct evidence for cGAS-STING engagement by kaempferol in pancreatic tissue is currently lacking (Wen et al., 2024).

Rosavin, the primary bioactive compound of Rhodiola rosea, exhibits multiple pharmacological effects, comprising anti-inflammatory, hepatoprotective properties and anti-adipogenic, making it particularly relevant for the treatment of metabolic syndrome-related diseases (Chen et al., 2022). Its mechanism of action may involve the inhibition of the NF-κB pathway, reduction of cell death, suppression of adipogenesis, and regulation of miRNA expression (Pu et al., 2020). This can illustrate that miRNAs may represent a potential molecular target of rosavin. Although no direct studies on acute pancreatitis (AP) have been reported to date, existing research indicates that in non-alcoholic fatty pancreas disease (NAFP), metabolic stress factors can trigger acinar cell injury, leading to progressive cell death and the development of AP. In a nonalcoholic fatty pancreas model, benzyl propylene glycoside, a distinct compound, has been reported to modulate cGAS-STING signaling and ameliorate pancreatic inflammation and fibrosis (Albadawy et al., 2023). However, whether rosavin shares this activity remains to be determined, and no direct evidence currently links rosavin to cGAS-STING modulation in pancreatic tissue.

Bufalin (BF), a potent cardiotonic steroid isolated from the skin and glands of toads (Soumoy et al., 2022), has attracted attention due to its diverse pharmacological properties, including analgesic, anti-tumor, and anti-inflammatory effects. Research has demonstrated that BF can attenuate inflammation and oxidative stress in experimental models of severe acute pancreatitis by activating the Keap1-Nrf2/HO-1 pathway and inhibiting the NF-κB pathway. Although bufalin has been reported to activate Keap1-Nrf2/HO-1 and inhibit NF-κB in experimental AP, direct evidence that bufalin modulates cGAS-STING signaling in pancreatic tissue is lacking. The cited studies (Long et al., 2022; Chen et al., 2023; Ding et al., 2023) were performed in gastric cancer, neuropathic pain, and acute kidney injury models, respectively, and therefore provide only cross-system, hypothesis-generating evidence. Future studies are needed to determine whether bufalin influences cGAS-STING in AP.

Through the above paper, we compared several drugs in a side-by-side manner and reached the conclusion that critical assessment of natural compounds as cGAS-STING-targeted therapeutics requires consideration of pharmacokinetic constraints, pancreatic tissue exposure, and target selectivity.

Several limitations are evident: Saikosaponin D: Oral bioavailability is below 15% due to intestinal degradation, and its pharmacokinetic profile is further complicated by CYP and P-glycoprotein interactions. Pancreatic tissue distribution data are lacking. Rosavin: Oral bioavailability is 4.7%, with poor membrane permeability and significant first-pass metabolism. Kaempferol: While kaempferol has demonstrated target engagement with STING/NLRP3 in adipose and pulmonary tissues, its pancreatic distribution and target selectivity profile have not been characterized. Bufalin: Although bufalin has demonstrated efficacy in SAP models, its narrow therapeutic window—as a cardiotonic steroid—raises safety concerns that require formal dose-ranging and toxicological evaluation.

## 7 Conclusion

Innate immunity plays a central role in the pathogenesis of acute pancreatitis. Dysregulated innate immune responses can exacerbate acinar cell injury and are closely associated with the development of chronic pancreatitis and pancreatic cancer. As a key cytosolic DNA sensor, the cGAS-STING pathway occupies a pivotal position in the innate immune response. Although the DNA sensor cGAS can detect self-DNA released from damaged cells or tissues, excessive activation of this pathway may trigger aberrant pancreatic inflammatory responses and contribute to disease progression. Recent studies on the cGAS-STING pathway in AP have significantly advanced our understanding of how innate immunity influences the initiation and progression of pancreatic inflammation and tissue injury. Emerging insights into the regulation of this pathway offer promising opportunities for identifying key molecular targets for therapeutic intervention in acute pancreatitis. The role of cGAS-STING in PSCs and pancreatic fibrosis warrants dedicated investigation in future reviews.

Indeed, the cGAS-STING pathway exhibits a dual functional role, often described as a “double-edged sword”. The therapeutic window for STING modulation in AP is constrained by the pathway’s dual role in host defense and inflammation. Transient STING activation is essential for pathogen clearance and tissue repair, whereas sustained or excessive activation drives pancreatic necrosis and systemic inflammation. This narrow therapeutic index raises the critical question of whether STING inhibition should be partial and time-limited rather than complete and sustained. The natural compounds discussed in this review largely lack systematic characterization of target selectivity, dose-response relationships, and pancreatic tissue exposure. For any natural compound to be considered a viable STING-targeted therapeutic for AP, the following evidence would be required: (1) demonstration of target engagement with cGAS or STING in pancreatic cells; (2) pharmacokinetic data confirming therapeutic concentrations in pancreatic tissue; (3) selectivity profiling against other innate immune sensors; and (4) safety evaluation in relevant AP models. At present, none of the compounds reviewed herein meets these criteria. Accordingly, this will constitute a major focus of our future mechanistic investigations into this class of natural compounds.

## References

[B1] Akazawa S. Mackin L. Jhala G. Fynch S. Catterall T. Selck C. . (2021). Deficiency of the innate immune adaptor STING promotes autoreactive T cell expansion in NOD mice. Diabetologia 64, 878–889. doi: 10.1007/s00125-020-05378-z 33483762

[B2] Albadawy R. Hasanin A. H. Agwa S. H. A. Hamady S. Mohamed R. H. Gomaa E. . (2023). Prospective insight into the role of benzyl propylene glycoside as a modulator of the cGAS-STING signaling pathway in the management of nonalcoholic fatty pancreas animal model. Biol. Res. 56, 11. doi: 10.1186/s40659-023-00423-8 36915161 PMC10010022

[B3] Aney K. J. Jeong W.-J. Vallejo A. F. Burdziak C. Chen E. Wang A. . (2024). Novel approach for pancreas transcriptomics reveals the cellular landscape in homeostasis and acute pancreatitis. Gastroenterology 166, 1100–1113. doi: 10.1053/j.gastro.2024.01.043 38325760 PMC11102849

[B4] Antonucci L. Fagman J. B. Kim J. Y. Todoric J. Gukovsky I. Mackey M. . (2015). Basal autophagy maintains pancreatic acinar cell homeostasis and protein synthesis and prevents ER stress. Proc. Natl. Acad. Sci. U.S.A. 112, E6166–E6174. doi: 10.1073/pnas.1519384112 26512112 PMC4653219

[B5] Balka K. R. Louis C. Saunders T. L. Smith A. M. Calleja D. J. D’Silva D. B. . (2020). TBK1 and IKKϵ act redundantly to mediate STING-induced NF-κB responses in myeloid cells. Cell Rep. 31, 107492. doi: 10.1016/j.celrep.2020.03.056 32268090

[B6] Banerjee I. Behl B. Mendonca M. Shrivastava G. Russo A. J. Menoret A. . (2018). Gasdermin D restrains type I interferon response to cytosolic DNA by disrupting ionic homeostasis. Immunity 49, 413–426.e5. doi: 10.1016/j.immuni.2018.07.006 30170814 PMC6347470

[B7] Bartok E. Hartmann G. (2020). Immune sensing mechanisms that discriminate self from altered self and foreign nucleic acids. Immunity 53, 54–77. doi: 10.1016/j.immuni.2020.06.014 32668228 PMC7359798

[B8] Bedrosian A. S. Nguyen A. H. Hackman M. Connolly M. K. Malhotra A. Ibrahim J. . (2011). Dendritic cells promote pancreatic viability in mice with acute pancreatitis. Gastroenterology 141, 1915–1926.e1-14. doi: 10.1053/j.gastro.2011.07.033 21801698 PMC3202684

[B9] Benninger R. K. P. Kravets V. (2022). The physiological role of β-cell heterogeneity in pancreatic islet function. Nat. Rev. Endocrinol. 18, 9–22. doi: 10.1038/s41574-021-00568-0 34667280 PMC8915749

[B10] Chen J. Li F. Luo W.-S. Zhu M.-F. Zhao N.-J. Zhang Z.-H. . (2025). Therapeutic potential of Da Cheng Qi decoction and its ingredients in regulating ferroptosis via the NOX2-GPX4 signaling pathway to alleviate and predict severe acute pancreatitis. Cell. Signal. 131, 111733. doi: 10.1016/j.cellsig.2025.111733 40081545

[B11] Chen H. Lu X. Xu B. Cheng G. Li Y. Xie D. (2024). Saikosaponin d protects pancreatic acinar cells against cerulein-induced pyroptosis through alleviating mitochondrial damage and inhibiting cGAS-STING pathway. J. Appl. Toxicol. 44, 1005–1013. doi: 10.1002/jat.4594 38462915

[B12] Chen Q. Sun L. Chen Z. J. (2016). Regulation and function of the cGAS-STING pathway of cytosolic DNA sensing. Nat. Immunol. 17, 1142–1149. doi: 10.1038/ni.3558 27648547

[B13] Chen H. Sun H. You F. Sun W. Zhou X. Chen L. . (2011). Activation of STAT6 by STING is critical for antiviral innate immunity. Cell. 147, 436–446. doi: 10.1016/j.cell.2011.09.022 22000020

[B14] Chen Y. Tang M. Yuan S. Fu S. Li Y. Li Y. . (2022). Rhodiola rosea: a therapeutic candidate on cardiovascular diseases. Oxid. Med. Cell. Longev. 2022, 1348795. doi: 10.1155/2022/1348795 35265260 PMC8898776

[B15] Chen L. Zhang X. Liu Y. Liu L. Liang X. Yang S. . (2023). JMJD3 is required for acute pancreatitis and pancreatitis-associated lung injury. J. Immunol. 210, 180–190. doi: 10.4049/jimmunol.2200484 36458991 PMC9772398

[B16] Chen G. Zhang H. Sun H. Ding X. Liu G. Yang F. . (2023). Bufalin targeting BFAR inhibits the occurrence and metastasis of gastric cancer through PI3K/AKT/mTOR signal pathway. Apoptosis 28, 1390–1405. doi: 10.1007/s10495-023-01855-z 37253905

[B17] Chen R. Zou J. Chen J. Zhong X. Kang R. Tang D. (2025). Pattern recognition receptors: Function, regulation and therapeutic potential. Signal. Transduct Target Ther. 10, 216. doi: 10.1038/s41392-025-02264-1 40640149 PMC12246121

[B18] Cheng Z. Dai T. He X. Zhang Z. Xie F. Wang S. . (2020). The interactions between cGAS-STING pathway and pathogens. Signal. Transduct Target Ther. 5, 91. doi: 10.1038/s41392-020-0198-7 32532954 PMC7293265

[B19] Chooklin S. Chuklin S. (2026). Nanotherapy for acute pancreatitis: a systematic review of experimental strategies and mechanisms of action. Discover Nano 21, 37. doi: 10.1186/s11671-026-04458-0 41661463 PMC12886637

[B20] Civril F. Deimling T. de Oliveira Mann C. C. Ablasser A. Moldt M. Witte G. . (2013). Structural mechanism of cytosolic DNA sensing by cGAS. Nature 498, 332–337. doi: 10.1038/nature12305 23722159 PMC3768140

[B21] Corrales L. McWhirter S. M. Dubensky T. W. Gajewski T. F. (2016). The host STING pathway at the interface of cancer and immunity. J. Clin. Invest. 126, 2404–2411. doi: 10.1172/JCI86892 27367184 PMC4922692

[B22] Cosentino K. Hertlein V. Jenner A. Dellmann T. Gojkovic M. Peña-Blanco A. . (2022). The interplay between BAX and BAK tunes apoptotic pore growth to control mitochondrial-DNA-mediated inflammation. Mol. Cell 82, 933–949.e9. doi: 10.1016/j.molcel.2022.01.008 35120587 PMC8901441

[B23] Criddle D. N. McLaughlin E. Murphy J. A. Petersen O. H. Sutton R. (2007). The pancreas misled: Signals to pancreatitis. Pancreatology 7, 436–446. doi: 10.1159/000108960 17898533

[B24] Dai E. Han L. Liu J. Xie Y. Zeh H. J. Kang R. . (2020). Ferroptotic damage promotes pancreatic tumorigenesis through a TMEM173/STING-dependent DNA sensor pathway. Nat. Commun. 11, 6339. doi: 10.1038/s41467-020-20154-8 33311482 PMC7732843

[B25] Del Poggetto E. Ho I.-L. Balestrieri C. Yen E.-Y. Zhang S. Citron F. . (2021). Epithelial memory of inflammation limits tissue damage while promoting pancreatic tumorigenesis. Science 373, eabj0486. doi: 10.1126/science.abj0486 34529467 PMC9733946

[B26] Diakopoulos K. N. Lesina M. Wörmann S. Song L. Aichler M. Schild L. . (2015). Impaired autophagy induces chronic atrophic pancreatitis in mice via sex- and nutrition-dependent processes. Gastroenterology 148, 626–638.e17. doi: 10.1053/j.gastro.2014.12.003 25497209

[B27] Ding L. Yin J. Xu X. Xie D. Xiang D. Tong P. . (2023). Bufalin alleviates acute kidney injury by regulating NLRP3 inflammasome-mediated pyroptosis. Apoptosis 28, 539–548. doi: 10.1007/s10495-023-01815-7 36652129

[B28] Du M. Chen Z. J. (2018). DNA-induced liquid phase condensation of cGAS activates innate immune signaling. Science 361, 704–709. doi: 10.1126/science.aat1022 29976794 PMC9417938

[B29] Dvorkin S. Cambier S. Volkman H. E. Stetson D. B. (2024). New frontiers in the cGAS-STING intracellular DNA-sensing pathway. Immunity 57, 718–730. doi: 10.1016/j.immuni.2024.02.019 38599167 PMC11013568

[B30] Fang Z. Li J. Cao F. Li F. (2022). Integration of scRNA-Seq and bulk RNA-Seq reveals molecular characterization of the immune microenvironment in acute pancreatitis. Biomolecules 13, 78. doi: 10.3390/biom13010078 36671463 PMC9855877

[B31] Ferrero-Andrés A. Panisello-Roselló A. Roselló-Catafau J. Folch-Puy E. (2020). NLRP3 inflammasome-mediated inflammation in acute pancreatitis. Int. J. Mol. Sci. 21, 5386. doi: 10.3390/ijms21155386 32751171 PMC7432368

[B32] Fink S. L. Cookson B. T. (2006). Caspase-1-dependent pore formation during pyroptosis leads to osmotic lysis of infected host macrophages. Cell. Microbiol. 8, 1812–1825. doi: 10.1111/j.1462-5822.2006.00751.x 16824040

[B33] Gall A. Treuting P. Elkon K. B. Loo Y.-M. Gale M. Barber G. N. . (2012). Autoimmunity initiates in nonhematopoietic cells and progresses via lymphocytes in an interferon-dependent autoimmune disease. Immunity 36, 120–131. doi: 10.1016/j.immuni.2011.11.018 22284419 PMC3269499

[B34] Gao Y. Mi N. Wu W. Zhao Y. Fan F. Liao W. . (2024). Transfer of inflammatory mitochondria via extracellular vesicles from M1 macrophages induces ferroptosis of pancreatic beta cells in acute pancreatitis. J. Extracell. Vesicles 13, e12410. doi: 10.1002/jev2.12410 38320981 PMC10847061

[B35] Gomez G. Lee H. M. He Q. Englander E. W. Uchida T. Greeley G. H. (2001). Acute pancreatitis signals activation of apoptosis-associated and survival genes in mice. Exp. Biol. Med. (Maywood) 226, 692–700. doi: 10.1177/153537020222600716 11444106

[B36] Gukovskaya A. S. Pandol S. J. Gukovsky I. (2016). New insights into the pathways initiating and driving pancreatitis. Curr. Opin. Gastroenterol. 32, 429–435. doi: 10.1097/MOG.0000000000000301 27428704 PMC5235997

[B37] Gukovsky I. Li N. Todoric J. Gukovskaya A. Karin M. (2013). Inflammation, autophagy, and obesity: Common features in the pathogenesis of pancreatitis and pancreatic cancer. Gastroenterology 144, 1199–1209.e4. doi: 10.1053/j.gastro.2013.02.007 23622129 PMC3786712

[B38] Gutierrez M. G. Master S. S. Singh S. B. Taylor G. A. Colombo M. I. Deretic V. (2004). Autophagy is a defense mechanism inhibiting BCG and Mycobacterium tuberculosis survival in infected macrophages. Cell. 119, 753–766. doi: 10.1016/j.cell.2004.11.038 15607973

[B39] Habtezion A. (2015). Inflammation in acute and chronic pancreatitis. Curr. Opin. Gastroenterol. 31, 395–399. doi: 10.1097/MOG.0000000000000195 26107390 PMC4618697

[B40] Habtezion A. Gukovskaya A. S. Pandol S. J. (2019). Acute pancreatitis: A multifaceted set of organelle and cellular interactions. Gastroenterology 156, 1941–1950. doi: 10.1053/j.gastro.2018.11.082 30660726 PMC6613790

[B41] Hashimoto D. Ohmuraya M. Hirota M. Yamamoto A. Suyama K. Ida S. . (2008). Involvement of autophagy in trypsinogen activation within the pancreatic acinar cells. J. Cell Biol. 181, 1065–1072. doi: 10.1083/jcb.200712156 18591426 PMC2442206

[B42] Helin H. Mero M. Markkula H. Helin M. (1980). Pancreatic acinar ultrastructure in human acute pancreatitis. Virchows Arch. A. Pathol. Anat. Histol. 387, 259–270. doi: 10.1007/BF00454829 7456314

[B43] Hopfner K.-P. Hornung V. (2020). Molecular mechanisms and cellular functions of cGAS-STING signalling. Nat. Rev. Mol. Cell Biol. 21, 501–521. doi: 10.1038/s41580-020-0244-x 32424334

[B44] Hou Y. Liang H. Rao E. Zheng W. Huang X. Deng L. . (2018). Non-canonical NF-κB antagonizes STING sensor-mediated DNA sensing in radiotherapy. Immunity 49, 490–503.e4. doi: 10.1016/j.immuni.2018.07.008 30170810 PMC6775781

[B45] Hu H. Q. Qiao J. T. Liu F. Q. Wang J. B. Sha S. He Q. . (2020). The STING-IRF3 pathway is involved in lipotoxic injury of pancreatic β cells in type 2 diabetes. Mol. Cell. Endocrinol. 518, 110890. doi: 10.1016/j.mce.2020.110890 32781250

[B46] Huang C. Xie S. Liu Y. Li Y. Lan G. He C. (2026). The pathogenesis and therapies of acute pancreatitis: A bibliometric analysis from 2003 to 2025. J. Inflammation Res. 19, 598878. doi: 10.2147/JIR.S598878 42205307 PMC13207949

[B47] Huang H. Lu W. Zhang X. Pan J. Cao F. Wen L. (2024). Fibroblast subtypes in pancreatic cancer and pancreatitis: From mechanisms to therapeutic strategies. Cell. Oncol. (Dordr) 47, 383–396. doi: 10.1007/s13402-023-00874-x 37721678 PMC12974079

[B48] Jia M. Qin D. Zhao C. Chai L. Yu Z. Wang W. . (2020). Redox homeostasis maintained by GPX4 facilitates STING activation. Nat. Immunol. 21, 727–735. doi: 10.1038/s41590-020-0699-0 32541831

[B49] Kimita W. Petrov M. S. (2020). Iron metabolism and the exocrine pancreas. Clin. Chim. Acta 511, 167–176. doi: 10.1016/j.cca.2020.10.013 33058846

[B50] King R. Hill J. L. Saha B. Tong Y. Strutt B. J. Russell M. A. . (2019). Offspring of mice exposed to a low-protein diet in utero demonstrate changes in mTOR signaling in pancreatic islets of Langerhans, associated with altered glucagon and insulin expression and a lower β-cell mass. Nutrients 11, 605. doi: 10.3390/nu11030605 30871106 PMC6471519

[B51] Kocsis A. K. Szabolcs A. Hofner P. Takács T. Farkas G. Boda K. . (2009). Plasma concentrations of high-mobility group box protein 1, soluble receptor for advanced glycation end-products and circulating DNA in patients with acute pancreatitis. Pancreatology 9, 383–391. doi: 10.1159/000181172 19451748

[B52] Kwon J. Bakhoum S. F. (2020). The cytosolic DNA-sensing cGAS-STING pathway in cancer. Cancer Discov. 10, 26–39. doi: 10.1158/2159-8290.CD-19-0761 31852718 PMC7151642

[B53] Kylänpää M.-L. Repo H. Puolakkainen P. A. (2010). Inflammation and immunosuppression in severe acute pancreatitis. World J. Gastroenterol. 16, 2867–2872. doi: 10.3748/wjg.v16.i23.2867 20556831 PMC2887581

[B54] Li C. Cui L. Zhang L. Yang L. Zhuo Y. Cui J. . (2021). Saikosaponin D attenuates pancreatic injury through suppressing the apoptosis of acinar cell via modulation of the MAPK signaling pathway. Front. Pharmacol. 12, 735079. doi: 10.3389/fphar.2021.735079 34744719 PMC8566544

[B55] Li P. Zheng Z. Qi J. Gao Y. Yang L. Li L. . (2023). HDAC3 improves intestinal function of mice by regulating cGAS-Sting pathway of intestinal glial cells. Mol. Immunol. 162, 95–101. doi: 10.1016/j.molimm.2023.08.012 37666082

[B56] Liang Q. Seo G. J. Choi Y. J. Kwak M.-J. Ge J. Rodgers M. A. . (2014). Crosstalk between the cGAS DNA sensor and Beclin-1 autophagy protein shapes innate antimicrobial immune responses. Cell Host Microbe 15, 228–238. doi: 10.1016/j.chom.2014.01.009 24528868 PMC3950946

[B57] Liu B. C. Sarhan J. Panda A. Muendlein H. I. Ilyukha V. Coers J. . (2018). Constitutive interferon maintains GBP expression required for release of bacterial components upstream of pyroptosis and anti-DNA responses. Cell Rep. 24, 155–168.e5. doi: 10.1016/j.celrep.2018.06.012 29972777 PMC6063733

[B58] Lohard S. Bourgeois N. Maillet L. Gautier F. Fétiveau A. Lasla H. . (2020). STING-dependent paracriny shapes apoptotic priming of breast tumors in response to anti-mitotic treatment. Nat. Commun. 11, 259. doi: 10.1038/s41467-019-13689-y 31937780 PMC6959316

[B59] Long L. Zhong W. Guo L. Ji J. Nie H. (2022). Effect of bufalin-PLGA microspheres in the alleviation of neuropathic pain via the CCI model. Front. Pharmacol. 13, 910885. doi: 10.3389/fphar.2022.910885 35770074 PMC9234216

[B60] Lopez-Font I. Gea-Sorlí S. de-Madaria E. Gutiérrez L. M. Pérez-Mateo M. Closa D. (2010). Pancreatic and pulmonary mast cells activation during experimental acute pancreatitis. World J. Gastroenterol. 16, 3411–3417. doi: 10.3748/wjg.v16.i27.3411 20632444 PMC2904888

[B61] Lu S. Wang P. Hu J. Zhang Z. (2025). Tetrandrine improves severe acute pancreatitis by inhibiting NCOA4 glycosylation-mediated binding to FTH1 and inducing autophagy-dependent ferroptosis. FASEB J. 39, e70592. doi: 10.1096/fj.202500404R 40326888

[B62] Lyu S. Liu S. Guo X. Zhang Y. Liu Z. Shi S. . (2024). hP-MSCs attenuate severe acute pancreatitis in mice via inhibiting NLRP3 inflammasome-mediated acinar cell pyroptosis. Apoptosis 29, 920–933. doi: 10.1007/s10495-024-01946-5 38625481

[B63] Mankan A. K. Schmidt T. Chauhan D. Goldeck M. Höning K. Gaidt M. . (2014). Cytosolic RNA:DNA hybrids activate the cGAS-STING axis. EMBO J. 33, 2937–2946. doi: 10.15252/embj.201488726 25425575 PMC4282641

[B64] Mareninova O. A. Hermann K. French S. W. O’Konski M. S. Pandol S. J. Webster P. . (2009). Impaired autophagic flux mediates acinar cell vacuole formation and trypsinogen activation in rodent models of acute pancreatitis. J. Clin. Invest. 119, 3340–3355. doi: 10.1172/JCI38674 19805911 PMC2769194

[B65] Martinon F. Burns K. Tschopp J. (2002). The inflammasome: a molecular platform triggering activation of inflammatory caspases and processing of proIL-beta. Mol. Cell 10, 417–426. doi: 10.1016/s1097-2765(02)00599-3 12191486

[B66] Mathur V. Burai R. Vest R. T. Bonanno L. N. Lehallier B. Zardeneta M. E. . (2017). Activation of the STING-dependent type I interferon response reduces microglial reactivity and neuroinflammation. Neuron 96, 1290–1302.e6. doi: 10.1016/j.neuron.2017.11.032 29268096 PMC5806703

[B67] Mayerle J. Sendler M. Hegyi E. Beyer G. Lerch M. M. Sahin-Tóth M. (2019). Genetics, cell biology, and pathophysiology of pancreatitis. Gastroenterology 156, 1951–1968.e1. doi: 10.1053/j.gastro.2018.11.081 30660731 PMC6903413

[B68] Mesev E. V. LeDesma R. A. Ploss A. (2019). Decoding type I and III interferon signalling during viral infection. Nat. Microbiol. 4, 914–924. doi: 10.1038/s41564-019-0421-x 30936491 PMC6554024

[B69] Mihoc T. Latcu S. C. Secasan C.-C. Dema V. Cumpanas A. A. Selaru M. . (2024). Pancreatic morphology, immunology, and the pathogenesis of acute pancreatitis. Biomedicines 12, 2627. doi: 10.3390/biomedicines12112627 39595191 PMC11591934

[B70] Mititelu A. Grama A. Colceriu M.-C. Benţa G. Popoviciu M.-S. Pop T. L. (2024). Role of interleukin 6 in acute pancreatitis: A possible marker for disease prognosis. Int. J. Mol. Sci. 25, 8283. doi: 10.3390/ijms25158283 39125854 PMC11311934

[B71] Mizushima N. Yamamoto A. Matsui M. Yoshimori T. Ohsumi Y. (2004). *In vivo* analysis of autophagy in response to nutrient starvation using transgenic mice expressing a fluorescent autophagosome marker. Mol. Biol. Cell 15, 1101–1111. doi: 10.1091/mbc.e03-09-0704 14699058 PMC363084

[B72] Ning X. Wang Y. Jing M. Sha M. Lv M. Gao P. . (2019). Apoptotic caspases suppress type I interferon production via the cleavage of cGAS, MAVS, and IRF3. Mol. Cell 74, 19–31.e7. doi: 10.1016/j.molcel.2019.02.013 30878284

[B73] Ning L. Wei W. Wenyang J. Rui X. Qing G. (2020). Cytosolic DNA-STING-NLRP3 axis is involved in murine acute lung injury induced by lipopolysaccharide. Clin. Transl. Med. 10, e228. doi: 10.1002/ctm2.228 33252860 PMC7668192

[B74] Nössing C. Ryan K. M. (2023). 50 years on and still very much alive: “Apoptosis: A basic biological phenomenon with wide-ranging implications in tissue kinetics. Br. J. Cancer 128, 426–431. doi: 10.1038/s41416-022-02020-0 36369364 PMC9938139

[B75] Ohmuraya M. Yamamura K. (2008). Autophagy and acute pancreatitis: A novel autophagy theory for trypsinogen activation. Autophagy 4, 1060–1062. doi: 10.4161/auto.6825 18776738

[B76] Peng Y. Yang Y. Li Y. Shi T. Xu N. Liu R. . (2024). Mitochondrial (Mt)DNA-cyclic GMP-AMP synthase (cGAS)-stimulator of interferon genes (STING) signaling promotes pyroptosis of macrophages via interferon regulatory factor (IRF)7/IRF3 activation to aggravate lung injury during severe acute pancreatitis. Cell. Mol. Biol. Lett. 29, 61. doi: 10.1186/s11658-024-00575-9 38671352 PMC11055249

[B77] Petrov M. S. Shanbhag S. Chakraborty M. Phillips A. R. J. Windsor J. A. (2010). Organ failure and infection of pancreatic necrosis as determinants of mortality in patients with acute pancreatitis. Gastroenterology 139, 813–820. doi: 10.1053/j.gastro.2010.06.010 20540942

[B78] Petrov M. S. Yadav D. (2019). Global epidemiology and holistic prevention of pancreatitis. Nat. Rev. Gastroenterol. Hepatol. 16, 175–184. doi: 10.1038/s41575-018-0087-5 30482911 PMC6597260

[B79] Priyanka Gupta S. Vatsa M. Mondal S . (2025). “ Regulators of system xc-–glutathione-glutathione peroxidase 4 antioxidant pathway: Recent advances and challenges in targeting ferroptosis for cancer treatment,” in Drug Discovery Today. 30(12), 104546. doi: 10.1016/j.drudis.2025.104546 41241377

[B80] Pu W.-L. Zhang M.-Y. Bai R.-Y. Sun L.-K. Li W.-H. Yu Y.-L. . (2020). Anti-inflammatory effects of Rhodiola rosea L.: a review. Biomed. Pharmacother. 121, 109552. doi: 10.1016/j.biopha.2019.109552 31715370

[B81] Qiu L. Ge L. Hu Q. (2020). Dexmedetomidine protects SK-N-SH nerve cells from oxidative injury by maintaining iron homeostasis. Biol. Pharm. Bull. 43, 424–431. doi: 10.1248/bpb.b19-00711 31839625

[B82] Ramirez M. L. G. Poreba M. Snipas S. J. Groborz K. Drag M. Salvesen G. S. (2018). Extensive peptide and natural protein substrate screens reveal that mouse caspase-11 has much narrower substrate specificity than caspase-1. J. Biol. Chem. 293, 7058–7067. doi: 10.1074/jbc.RA117.001329 29414788 PMC5936834

[B83] Riley J. S. Quarato G. Cloix C. Lopez J. O’Prey J. Pearson M. . (2018). Mitochondrial inner membrane permeabilisation enables mtDNA release during apoptosis. EMBO J. 37, e99238. doi: 10.15252/embj.201899238 30049712 PMC6120664

[B84] Sendler M. Mayerle J. Lerch M. M. (2016). Necrosis, apoptosis, necroptosis, pyroptosis: It matters how acinar cells die during pancreatitis. Cell. Mol. Gastroenterol. Hepatol. 2, 407–408. doi: 10.1016/j.jcmgh.2016.05.007 28174728 PMC5042603

[B85] Sendler M. Weiss F.-U. Golchert J. Homuth G. van den Brandt C. Mahajan U. M. . (2018). Cathepsin B-mediated activation of trypsinogen in endocytosing macrophages increases severity of pancreatitis in mice. Gastroenterology 154, 704–718.e10. doi: 10.1053/j.gastro.2017.10.018 29079517 PMC6663074

[B86] Shifrin A. L. Chirmule N. Gao G.-P. Wilson J. M. Raper S. E. (2005). Innate immune responses to adenoviral vector-mediated acute pancreatitis. Pancreas 30, 122–129. doi: 10.1097/01.mpa.0000151578.99413.88 15714134

[B87] Singh R. Letai A. Sarosiek K. (2019). Regulation of apoptosis in health and disease: The balancing act of BCL-2 family proteins. Nat. Rev. Mol. Cell Biol. 20, 175–193. doi: 10.1038/s41580-018-0089-8 30655609 PMC7325303

[B88] Son A. Ahuja M. Schwartz D. M. Varga A. Swaim W. Kang N. . (2019). Ca2+ influx channel inhibitor SARAF protects mice from acute pancreatitis. Gastroenterology 157, 1660–1672.e2. doi: 10.1053/j.gastro.2019.08.042 31493399

[B89] Song Y. Zhang Z. Yu Z. Xia G. Wang Y. Wang L. . (2021). Wip1 aggravates the cerulein-induced cell autophagy and inflammatory injury by targeting STING/TBK1/IRF3 in acute pancreatitis. Inflammation 44, 1175–1183. doi: 10.1007/s10753-021-01412-3 33417178

[B90] Soumoy L. Ghanem G. E. Saussez S. Journe F. (2022). Bufalin for an innovative therapeutic approach against cancer. Pharmacol. Res. 184, 106442. doi: 10.1016/j.phrs.2022.106442 36096424

[B91] Strum W. B. Boland C. R. (2023). Advances in acute and chronic pancreatitis. World J. Gastroenterol. 29, 1194–1201. doi: 10.3748/wjg.v29.i7.1194 36926670 PMC10011955

[B92] Szatmary P. Grammatikopoulos T. Cai W. Huang W. Mukherjee R. Halloran C. . (2022). Acute pancreatitis: Diagnosis and treatment. Drugs 82, 1251–1276. doi: 10.1007/s40265-022-01766-4 36074322 PMC9454414

[B93] Tang D. Kang R. Berghe T. V. Vandenabeele P. Kroemer G. (2019). The molecular machinery of regulated cell death. Cell Res. 29, 347–364. doi: 10.1038/s41422-019-0164-5 30948788 PMC6796845

[B94] Tenner S. Vege S. S. Sheth S. G. Sauer B. Yang A. Conwell D. L. . (2024). American College of Gastroenterology guidelines: Management of acute pancreatitis. Am. J. Gastroenterol. 119, 419–437. doi: 10.14309/ajg.0000000000002645 38857482 PMC13221274

[B95] Trikudanathan G. Yazici C. Evans Phillips A. Forsmark C. E. (2024). Diagnosis and management of acute pancreatitis. Gastroenterology 167, 673–688. doi: 10.1053/j.gastro.2024.02.052 38759844

[B96] Voet S. Srinivasan S. Lamkanfi M. van Loo G. (2019). Inflammasomes in neuroinflammatory and neurodegenerative diseases. EMBO Mol. Med. 11, e10248. doi: 10.15252/emmm.201810248 31015277 PMC6554670

[B97] Wan J. Yang X. Ren Y. Li X. Zhu Y. Haddock A. N. . (2019). Inhibition of miR-155 reduces impaired autophagy and improves prognosis in an experimental pancreatitis mouse model. Cell Death Dis. 10, 303. doi: 10.1038/s41419-019-1545-x 30944299 PMC6447551

[B98] Wang X. Chen T. Chen S. Zhang J. Cai L. Liu C. . (2025). STING aggravates ferroptosis-dependent myocardial ischemia-reperfusion injury by targeting GPX4 for autophagic degradation. Sig Transduct Target Ther. 10, 136. doi: 10.1038/s41392-025-02216-9 PMC12022026 40274801

[B99] Wang H. Gao J. Wen L. Huang K. Liu H. Zeng L. . (2024). Ion channels in acinar cells in acute pancreatitis: Crosstalk of calcium, iron, and copper signals. Front. Immunol. 15, 1444272. doi: 10.3389/fimmu.2024.1444272 39606246 PMC11599217

[B100] Wang Z. Liu J. Li F. Luo Y. Ge P. Zhang Y. . (2022). The gut-lung axis in severe acute pancreatitis-associated lung injury: the protection by the gut microbiota through short-chain fatty acids. Pharmacol. Res. 182, 106321. doi: 10.1016/j.phrs.2022.106321 35752356

[B101] Wang Z. Liu J. Wang Y. Guo H. Li F. Cao Y. . (2023). Identification of key biomarkers associated with immunogenic cell death and their regulatory mechanisms in severe acute pancreatitis based on WGCNA and machine learning. Int. J. Mol. Sci. 24, 3033. doi: 10.3390/ijms24033033 36769358 PMC9918120

[B102] Wang Y. Ning X. Gao P. Wu S. Sha M. Lv M. . (2017). Inflammasome activation triggers caspase-1-mediated cleavage of cGAS to regulate responses to DNA virus infection. Immunity 46, 393–404. doi: 10.1016/j.immuni.2017.02.011 28314590

[B103] Watanabe T. Kudo M. Strober W. (2017). Immunopathogenesis of pancreatitis. Mucosal Immunol. 10, 283–298. doi: 10.1038/mi.2016.101 27848953

[B104] Wei H. Chen Y. Qin Z. Wang H. Liu Y. Song T. . (2025). Artesunate demonstrates neuroprotective effect through activation of lysosomal function and inhibition of cGAS-STING pathway. Neuropharmacology 272, 110426. doi: 10.1016/j.neuropharm.2025.110426 40118208

[B105] Wen E. Cao Y. He S. Zhang Y. You L. Wang T. . (2024). The mitochondria-targeted kaempferol nanoparticle ameliorates severe acute pancreatitis. J. Nanobiotechnology 22, 148. doi: 10.1186/s12951-024-02439-y 38570776 PMC10993609

[B106] White M. J. McArthur K. Metcalf D. Lane R. M. Cambier J. C. Herold M. J. . (2014). Apoptotic caspases suppress mtDNA-induced STING-mediated type I IFN production. Cell. 159, 1549–1562. doi: 10.1016/j.cell.2014.11.036 25525874 PMC4520319

[B107] Wu J. Chen Y.-J. Dobbs N. Sakai T. Liou J. Miner J. J. . (2019). STING-mediated disruption of calcium homeostasis chronically activates ER stress and primes T cell death. J. Exp. Med. 216, 867–883. doi: 10.1084/jem.20182192 30886058 PMC6446864

[B108] Wu M.-Y. Lu J.-H. (2019). Autophagy and macrophage functions: Inflammatory response and phagocytosis. Cells 9, 70. doi: 10.3390/cells9010070 31892110 PMC7016593

[B109] Wu X. Zhao K. Fang X. Lu F. Cheng P. Song X. . (2022). Saikosaponin D inhibited IL-1β induced ATDC 5 chondrocytes apoptosis *in vitro* and delayed articular cartilage degeneration in OA model mice *in vivo*. Front. Pharmacol. 13, 845959. doi: 10.3389/fphar.2022.845959 35370642 PMC8975252

[B110] Xia S. Ruan J. Wu H. (2019). Monitoring gasdermin pore formation *in vitro*. Methods Enzymol. 625, 95–107. doi: 10.1016/bs.mie.2019.04.024 31455540 PMC7533106

[B111] Xia L. Xu Z. Zhou X. Bergmann F. Grabe N. Büchler M. W. . (2020). Impaired autophagy increases susceptibility to endotoxin-induced chronic pancreatitis. Cell Death Dis. 11, 889. doi: 10.1038/s41419-020-03050-3 33087696 PMC7578033

[B112] Ye R. Onodera T. Scherer P. E. (2019). Lipotoxicity and β cell maintenance in obesity and type 2 diabetes. J. Endocr. Soc 3, 617–631. doi: 10.1210/js.2018-00372 30834357 PMC6391718

[B113] Zhang X. Bai X.-C. Chen Z. J. (2020). Structures and mechanisms in the cGAS-STING innate immunity pathway. Immunity 53, 43–53. doi: 10.1016/j.immuni.2020.05.013 32668227

[B114] Zhang T. Chen S. Li L. Jin Y. Liu S. Liu Z. . (2024). PFKFB3 controls acinar IP3R-mediated Ca2+ overload to regulate acute pancreatitis severity. JCI Insight 9, e169481. doi: 10.1172/jci.insight.169481 38781030 PMC11383365

[B115] Zhang Y. Jiang Y. Li H. Wang J. Li C. Zhang D. (2023). Effect of STING signaling on intestinal barrier damage in severe acute pancreatitis. Exp. Cell. Res. 428, 113630. doi: 10.1016/j.yexcr.2023.113630 37196844

[B116] Zhang D. Li J. Zhao L. Yang Z. Wu C. Liu Y. . (2025). Mitochondrial DNA leakage promotes persistent pancreatic acinar cell injury in acute pancreatitis via the cGAS-STING-NF-κB pathway. Inflammation 48, 1420–1437. doi: 10.1007/s10753-024-02132-0 39180578

[B117] Zhang L. Wei X. Wang Z. Liu P. Hou Y. Xu Y. . (2023). NF-κB activation enhances STING signaling by altering microtubule-mediated STING trafficking. Cell Rep. 42, 112185. doi: 10.1016/j.celrep.2023.112185 36857187

[B118] Zhang B. Xu P. Ablasser A. (2025). Regulation of the cGAS-STING pathway. Annu. Rev. Immunol. 43, 667–692. doi: 10.1146/annurev-immunol-101721-032910 40085836

[B119] Zhao Q. Manohar M. Wei Y. Pandol S. J. Habtezion A. (2019). STING signalling protects against chronic pancreatitis by modulating Th17 response. Gut 68, 1827–1837. doi: 10.1136/gutjnl-2018-317098 30705050 PMC7069765

[B120] Zhao Q. Wei Y. Pandol S. J. Li L. Habtezion A. (2018a). STING signaling promotes inflammation in experimental acute pancreatitis. Gastroenterology 154, 1822–1835.e2. doi: 10.1053/j.gastro.2018.01.065 29425920 PMC6112120

[B121] Zhao Q. Wei Y. Pandol S. J. Li L. Habtezion A. (2018b). STING signaling promotes inflammation in experimental acute pancreatitis. Gastroenterology 154, 1822–1835.e2. doi: 10.1053/j.gastro.2018.01.065 29425920 PMC6112120

[B122] Zhao M. Xue D.-B. Zheng B. Zhang W.-H. Pan S.-H. Sun B. (2007). Induction of apoptosis by artemisinin relieving the severity of inflammation in caerulein-induced acute pancreatitis. World J. Gastroenterol. 13, 5612–5617. doi: 10.3748/wjg.v13.i42.5612 17948936 PMC4172741

[B123] Zheng W. Liu A. Xia N. Chen N. Meurens F. Zhu J. (2023). How the innate immune DNA sensing cGAS-STING pathway is involved in apoptosis. Int. J. Mol. Sci. 24, 3029. doi: 10.3390/ijms24033029 36769349 PMC9917431

[B124] Zhou Y. Huang X. Jin Y. Qiu M. Ambe P. C. Basharat Z. . (2024). The role of mitochondrial damage-associated molecular patterns in acute pancreatitis. Biomed. Pharmacother. 175, 116690. doi: 10.1016/j.biopha.2024.116690 38718519

[B125] Zhou Q. Tao X. Xia S. Guo F. Pan C. Xiang H. . (2020). T lymphocytes: A promising immunotherapeutic target for pancreatitis and pancreatic cancer? Front. Oncol. 10, 382. doi: 10.3389/fonc.2020.00382 32266154 PMC7105736

[B126] Zierhut C. Yamaguchi N. Paredes M. Luo J.-D. Carroll T. Funabiki H. (2019). The cytoplasmic DNA sensor cGAS promotes mitotic cell death. Cell. 178, 302–315.e23. doi: 10.1016/j.cell.2019.05.035 31299200 PMC6693521

